# The Accumulation of Deficits with Age and Possible Invariants of Aging

**DOI:** 10.1100/tsw.2002.861

**Published:** 2002-06-28

**Authors:** Arnold B. Mitnitski, Alexander J. Mogilner, Chris MacKnight, Kenneth Rockwood

**Affiliations:** Department of Medicine, Dalhousie University, 5955 Veterans’ Memorial Lane, Halifax, Nova Scotia, Canada B3H 2E1,

**Keywords:** aging, disability, frailty, frailty index, frailty trajectories, gender difference, compensation law, Gompertz-Makeham law, invariants of aging, biological constants, macroparameters, National Population Health Survey, databases, mathematical modeling

## Abstract

This paper extends a method of apprising health status to a broad range of ages from adolescence to old age. The “frailty index” is based on the accumulation of deficits (symptoms, signs, disease classifications) as analyzed in the National Population Health Survey, a representative Canadian population sample (n = 81,859). The accumulation of deficits has both an age-independent (background) component and an age-dependent (exponential) component, akin to the well-known Gompertz-Makeham model for the risk of mortality. While women accumulate more deficits than men of the same age, on average, their rate of accumulation is lower, so the difference in the level of deficits between men and women decreases with age. Two possible invariants of the process of accumulation of deficits were found: (1) the age at which the average proportion of deficits coincides for men and women is 94 years, which closely matches the *species-specific lifespan in humans* (95 ± 2); (2) the value of the frailty index (proportion of deficits), which corresponds to that age (0.18). The similarity between mortality kinetics and the accumulation of deficits (frailty kinetics), and the coincidence of the time parameters in the frailty and mortality models make it possible to express mortality risk in terms of accumulated deficits. This provides a simple and accessible tool that might have potential in a number of biomedical applications.

## INTRODUCTION

Older adults have varying states of health, but how to quantify average or individual differences is not clear[1,2,3]. We have examined the aging process as an accumulation of symptoms, signs, and disease classifications (jointly referred to as deficits) which, while age related, are not usually known as risks for diminished life expectancy, e.g., impaired vision or hearing, or back pain[1,2]. Those analyses proposed that a quantitative measure, the *frailty index* ($q$, herein referred to as $f$) based on the accumulation of such deficits, can be a means of assessing individual aging[1]. This index might serve as a practicable means of estimating differences in the individual health states of older adults. The frailty index is estimated as the proportion of deficits (coded simply as present/not present) detected in a given individual, and was demonstrated to be a group and individual indicator of health, and an important correlate of survival[1,2]. Those results, however, require replication; they were obtained by analyzing data from adults aged 65 years and older[4], they did not distinguish between sexes, and they were based on a comparatively small sample (n = 2,194). Now, using a large representative database (the Canadian National Population Health Survey - NPHS)[5], which contains tens of thousands of records across all age groups (though aggregated mainly by 5 year intervals), it is possible to extend our earlier analysis. We report the properties of the frailty index in this larger sample, and investigate additional insights about this method of aggregating individual health status.

## DATA

The data come from the cross-sectional component of the NPHS, an investigation of health, health status, and the use of health services in Canada. The database contains 81,859 records across all age groups (represented mainly by the five year intervals). The female/male ratio is 1.12. All 66,589 records of individuals aged 15 to 79 with complete records were used in the analysis; 38 binary (Yes/No) variables were available to characterize the health conditions (see Appendix). Items included symptoms (e.g., loss of vision), disabilities (dependence on someone else to prepare meals), and disease classifications (arthritis, Alzheimer's disease) jointly referred to as *deficits*. Note that the deficits cross a range of severity, from items associated with an increased risk of death (e.g., cancer) to those that typically cause more discomfort than disability (e.g., hearing problems). In this way, it is in contrast to, say, an activities of daily living (ADL) scale, i.e., it does not require the impairment to be severe enough to be disabling.

Each individual can be represented by a 38-dimension binary vector (an ordered sequence of 0s and 1s). As stated elsewhere[1], the proportion of deficits incurred by an individual can be considered as a measure of health status, namely the frailty index *(f)*. For example, an individual who reported having cataract, high blood pressure, and back pain (3 items) has a frailty index of 3/38 (0.08). Despite using a different set of deficits than that used previously[1,2], it will be demonstrated that the frailty index accumulates generally in the same fashion (exponentially). Gender differences in the accumulation of deficits are derived, but more importantly, common characteristics (invariants) of aging are revealed and quantified. Of note, the mortality rate can be expressed in terms of the accumulated deficits, and is better accounted for by variation in the accumulation of deficits than by variation in chronological age.

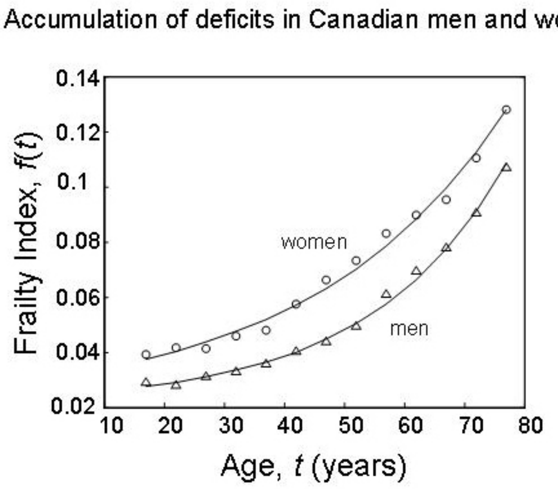

**FIGURE 1**. Accumulation of the proportion of deficits (frailty trajectories) with chronological age for men and women. Experimental data are the proportion of deficits average across the same age group. Age group is represented by the midpoint, e.g., from 50 to 55 years represented by the point at 52 years. Solid lines represent fitting curves according to the two-component model, Eq. 1.

## RESULTS AND DISCUSSION

### Sex Differences in the Accumulation of Deficits

In Fig.1, the proportion of deficits (frailty index) is presented as a function of age, $t$, for men and women and fit the following function:

$$f_i(t) = G_i + F_i \exp(\beta_i t) \quad (1)$$

(solid lines) with the parameters $G_i$ ,$F_i$ ,$\beta_i$ estimated from the data, ($i$ = 1 for men and 2 for women).

The least square estimates of the parameters were obtained as follows. The background parameter $G$ has the same value for males and females (0.02 ± 0.001). The parameters $F$ and $\beta$ were estimated as: $\ln(F_1) = -5.77 \pm 0.06$, $\beta_1 = 0.043 \pm 0.001$(1/year) in men, and $\ln(F_2) = -4.63 \pm 0.06$, $\beta_2 = 0.031 \pm 0.001$ (1/year) in women. On average, women have a greater value of their frailty index than men at the same age. According to Eq. 1, accumulation of deficits (frailty) may be represented by two terms: age independent, $G$ (background frailty) and age dependent, $F \exp(\beta t)$, similar to the Gompertz-Makeham law of mortality[6,7], $\mu = A + R \exp(\alpha t)$, where $\mu$ is the force of mortality, $A$ is background component of mortality, $R$ and $\alpha$ are the parameters of the age-dependent component of mortality. An extensive collection of the values of these parameters for different populations has been estimated[7]. This work demonstrates that the parameters $R$ and $\alpha$ have a reciprocal relationship, referred to as the *compensation law of mortality*[6,7,8]. This relationship explains the so-called Strehler-Mildvan correlation, which is observed if the background component $A$ is neglected[9]. Of some interest, the same type of relationship holds for the respective parameters ($F$, $\beta$) of the frailty model.

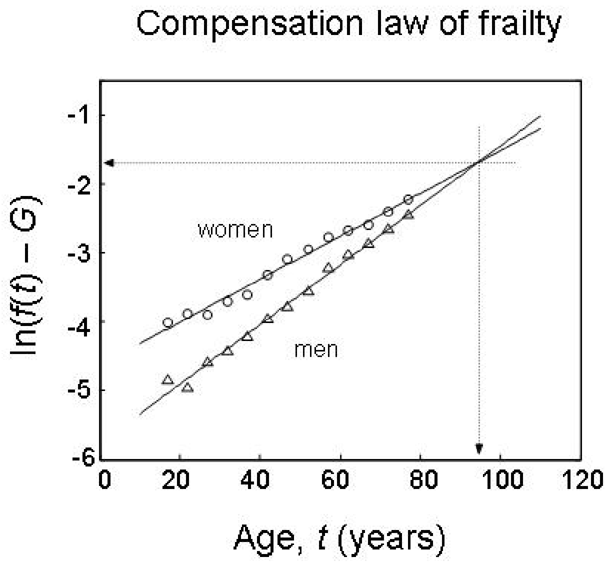

**FIGURE 2**. The compensation law of frailty. Logarithm of age-dependent component of frailty for men and women. The least square lines have a cross-point corresponding to the 0.18 of the frailty index at age of 94 years, the same age parameter for the compensation law of mortality[4,5].

## Compensation Law of Frailty and Its Possible Invariants

As one can see, the parameters $F$ and $\beta$ have a reciprocal relationship, similar to that reported for the mortality rate convergence[6] *(mortality compensation law*[8]): $\ln(R) = \ln(M) - aB$, where $M$ and $B$ are the *species-specific invariants* estimated in humans, 0.5 (1/year) and 95 years, respectively[6,7].

In Fig. 2, the logarithm of the age-dependent component of frailty is plotted against age. The straight lines closely represent the data (the correlation coefficients are 0.98 for men and 0.97 for women, corresponding to about 95% of the explained variance). The lines cross with the coordinates of abscissa, $T = 94 \pm 2$ years and ordinate, $H = 0.18 \pm 0.1$. By introducing $\ln(F) = \ln(H) - \beta_i T$ ($i$ =1,2), Eq. 1 may be presented as follows:

$$f_i(t) = G + H \exp[\beta_i (t - T)] \qquad (2)$$

The parameter $T$ has dimension of time. Of some interest, it equals the parameter $B$ found from mortality data[6,7], which has been termed the "*species-specific lifespan*" (95 ± 2 years in humans). The parameter $H$ is the proportion of deficits corresponding to the age, $T$, and is the specific proportion of deficits (frailty index). It is a dimensionless parameter, and possibly reflects the ability of the human organism to withstand damage. The only gender-dependent parameter is the rate $\beta_i$. Of note, the values of the rate parameters are close to those reported for the elderly Canadians (0.033 1/year) despite an essential difference in the deficits and the age scale in both studies[1,2].

That an estimate of the human-specific lifespan has been found from two different models seems to us to be quite remarkable. The Gompertz-Makeham law describes the dependence of the force of mortality on age, while Eq. 1 and 2 describe the *current state* of the system, in terms of accumulated deficits. We propose that can be taken to reflect the degree of damage of the system. Age is a common factor in both models and the fact that the age-dimension invariant is the same both in the dynamics of mortality and accumulation of deficits, would appear to us to indicate the unity of processes governing aging.

It has been recognized that "the problem of the individual differences in life time is indeed a key problem in the biology of life span"[7]. This problem can be addressed if mortality rate would be expressed as a function of accumulated deficits rather than chronological age. Comparing Eq. 2 with the Gompertz-Makeham law, the force of mortality $\mu$ may be expressed (after elementary transformations) as a function of deficits $f$:

$$\mu = A + M\,[(f - G)/H]^{\,\nu} \qquad (3)$$

where $\nu = \alpha/\beta$ is a ratio of the rate of age-dependent component of mortality to the rate of age-dependent component of accumulation deficits. Considering conditions in which the background components $A$ is small (typical for developed countries[6]), and neglecting a small value of $G$ compared to $f$, and taking into account estimates of invariants $M$ and $H$, one can arrive at the following formula:

$$\mu \approx 0.5\,(f/0.18)^{\,\nu} \qquad (4)$$

For the Canadian population[10] it can be estimated $\alpha \approx 0.118$ 1/year, hence $\nu = 2.74$ for men and 3.80 for women. For other countries the parameter $\nu$ might well be different (as its components, $\alpha$ and $\beta$) reflecting different conditions of life and health care in those countries.

## CONCLUSIONS

1. The principle of a frailty index has been cross-validated in this study, and extended to include a larger age range (17 to 79) with specific estimates for men and women.
2. Deficits (symptoms, disorders, disabilities) in individuals appear to accumulate according to exponential kinetics, similar to the Gompertz-Makeham mortality law.
3. We propose a *frailty compensation relationship,* which appears to be homologous (in that it demonstrates reciprocal relationships between the magnitude and exponent of the age-dependent component of the frailty index) to *the mortality compensation law,* itself established for many populations[6,7,8].
4. A possible invariant of aging, the *human-specific lifespan* has been estimated independently, and gives virtually the same value ($T = 94$ years) as its estimation in other contexts[6]. This indicates to us an essential relationship between the process of the accumulation of deficits and mortality kinetics. Another possible invariant estimated herein is the specific proportion of deficits corresponding to age, $T$ ($H = 0.18$).
5. The mortality rate can be expressed in terms of accumulated deficits. As such, the frailty index accounts for important individual differences between people of the same age, and therefore has potential to be used as in the assessment of individual health status.

## ACKNOWLEDGEMENT

This analysis was funded by Health Canada through the National Health Research and Development Program, Grant #6603-03-1999/2640043. Chris MacKnight is supported by a New Investigator Award and Kenneth Rockwood by an Investigator Award, each from the Canadian Institutes of Health Research. Kenneth Rockwood is also supported by the Dalhousie Medical Research Foundation as the Kathryn Allen Weldon Professor of Alzheimer Research.

## BIOSKETCH

**Arnold B. Mitnitski, Ph.D.,** is Assistant Professor, Department of Medicine, Dalhousie University. He holds an M.S. in Engineering and a Ph.D. in Dynamics and control processes. In general, his research interests lie in the area of mathematical modeling and computer simulation of complex biomedical systems. In particular, he is interested in studying the human organism as a complex dynamical system — what "macroscopic" variables (*state variables*) should be included in the outcome predictive model, what are the relationships between the signs, symptoms, and functional deficits in norm and pathologies. Modeling of biological aging is a particular area of recent interest. Other areas of interest are production of methodologies and environment, application of frameworks for knowledge discovery in databases (KDD) based on modern technologies (e.g., artificial intelligence) as well as "classical" approaches used in biophysics of complex systems.

## APPENDIX: LIST OF VARIABLES

Allergies (other than food allergies), asthma, arthritis or rheumatism, back pain (excluding arthritis), high blood pressure, migraine, chronic bronchitis, sinusitis, diabetes, epilepsy, heart disease, cancer, stomach or intestinal ulcers, suffering from the effects of a stroke, urinary incontinence, bowel disorder, Alzheimer's disease or other dementia, cataracts, glaucoma, thyroid condition, derived trouble with vision, hearing problems, speech, mobility, dexterity, emotional problems, cognition, activities prevented-pain/discomfort, severity of pain, needing help in preparing meals, shopping, housework, heavy household chores, moving in the house, needing to receive home care services.
